# Two-Drug Combinations Therapy of Different Doses of Valsartan Existing Diverse Significance for Hypertensive Patients

**DOI:** 10.31083/j.rcm2407187

**Published:** 2023-06-29

**Authors:** Zerong Wang, Shixiong Wang, Liqiong Zhang, Jiaxuan Wang, Rong Wang, Shude Chen, Qiling Shi, Hongye Wu, Liuyang Wang, Ningyin Li

**Affiliations:** ^1^The Second Clinical Medical College, Lanzhou University, 730000 Lanzhou, Gansu, China; ^2^Department of Cardiac Surgery, Lanzhou University Second Hospital, 730030 Lanzhou, Gansu, China; ^3^Cardiovascular Department, Lanzhou University Second Hospital, 730030 Lanzhou, Gansu, China

**Keywords:** hypertension, blood pressure, valsartan, combined therapy, clinical complications

## Abstract

**Background:**

The incidence of hypertension and clinical complications (e.g., heart, cerebrovascular and kidney injury) is increasing worldwide. It is widely known that a relatively large dose of valsartan (320 mg) could alleviate clinical complications. The current network meta-analysis assessed which drug could be combined with a relatively large dose of valsartan to control blood pressure (BP) more effectively. And which combination therapy with different dosages of valsartan did not induce excessive BP reduction with increasing dosages of valsartan.

**Methods:**

The PubMed, Embase, Medline, Cochrane Library, CNKI, Wanfang, and CSTJ databases were searched from inception to October 2022 for relevant randomized controlled trials (RCTs). The search strategies included concepts related to hypertension and two-drug combination therapy of different doses of valsartan, and there were no language or data restrictions. The outcomes included adverse effects and changes in systolic BP and diastolic BP. Permanent discontinuations related to treatment were the most accurate and objective measure of adverse effects. The common adverse effects of most studies (i.e., dizziness, headache, nasopharyngitis, asthenia and urticaria) were also included. A Bayesian network meta-analysis was performed, and mean differences with 95% confidence intervals were calculated. ADDIS and STATA were used for Bayesian model network meta-calculation.

**Results:**

Thirty-four RCTs were included involving 26,752 patients, and the interventions included different doses of valsartan combined with various types and doses of drugs. Among many combination therapies, the combination of valsartan 320 mg with amlodipine 10 mg (*p* < 0.01) had the best antihypertensive effect without significant adverse effects. Compared with valsartan 80 mg and 160 mg, valsartan 320 mg combined with hydrochlorothiazide 25 mg (*p* > 0.05) did not further reduce BP and was not shown to increase the incidence of adverse effects.

**Conclusions:**

Combination therapy with a relatively large dose of valsartan could control BP and improve clinical complications effectively. However, for hypertensive patients with different treatment requirements, specific choices should be made regarding whether to control BP, treat clinical complications, or both.

## 1. Introduction

Hypertension is a major risk factor for cardiovascular disease (CVD) and death worldwide [1]. The global burden of hypertension was approximately 1.4 billion in 2021 and may exceed 1.6 billion by 2025 [2]. The age-standardized prevalence of hypertension in adults aged 30–79 years was 33% in the global population [3]. At present, the number of CVD cases exceeds 500 million worldwide [4]. Additionally, the increasing incidence of hypertension and clinical complications (e.g., heart, cerebrovascular and kidney injury) has a serious impact on people’s health and quality of life. However, according to different studies, hypertension treatment and control rates are less than 50% and 20%, respectively [5, 6, 7]. The initial treatment recommended in recent research is antihypertensive treatment with combination therapy and the recommendation of single-pill combinations [8]. The use of drug combinations significantly decreases blood pressure (BP). In particular, combination therapy could improve clinical complications [9]. Numerous studies have indicated that renin-angiotensin system (RAS)-inhibiting drugs are the cornerstone of combination treatment for hypertension and are recommended for combination treatment [10]. The widely used fixed combination is based the addition of angiotensin II (Ang II) receptor blockers (ARBs), such as valsartan, to calcium channel blockers (CCBs) or thiazide diuretics [9]. In addition, the rate of adverse effects associated with the above combination treatment may be reduced because the effects of each agent are reciprocally counterbalanced [11]. Currently, although it is widely known that a relatively large dose of valsartan (320 mg) could treat clinical complications with relatively sufficient blocking of Ang II type 1 receptor (${\mathrm{AT}}_{1}$R), there is no clinical consensus regarding the influence of different dosages of valsartan combined with different types and dosages of other drugs on BP and clinical complications. Moreover, a single randomized controlled trial (RCT) and traditional meta-analysis could not provide strong evidence-based support. The purpose of the current network meta-analysis was to assess which drug could be combined with a relatively large dose of valsartan to more control BP more effectively. Combination therapy with valsartan could increase the dose of valsartan to a relatively large dose without causing an excessive reduction in BP.

## 2. Materials and Methods 

### 2.1 Search Strategy for Identifying Eligible Studies

**Fig. 1.**
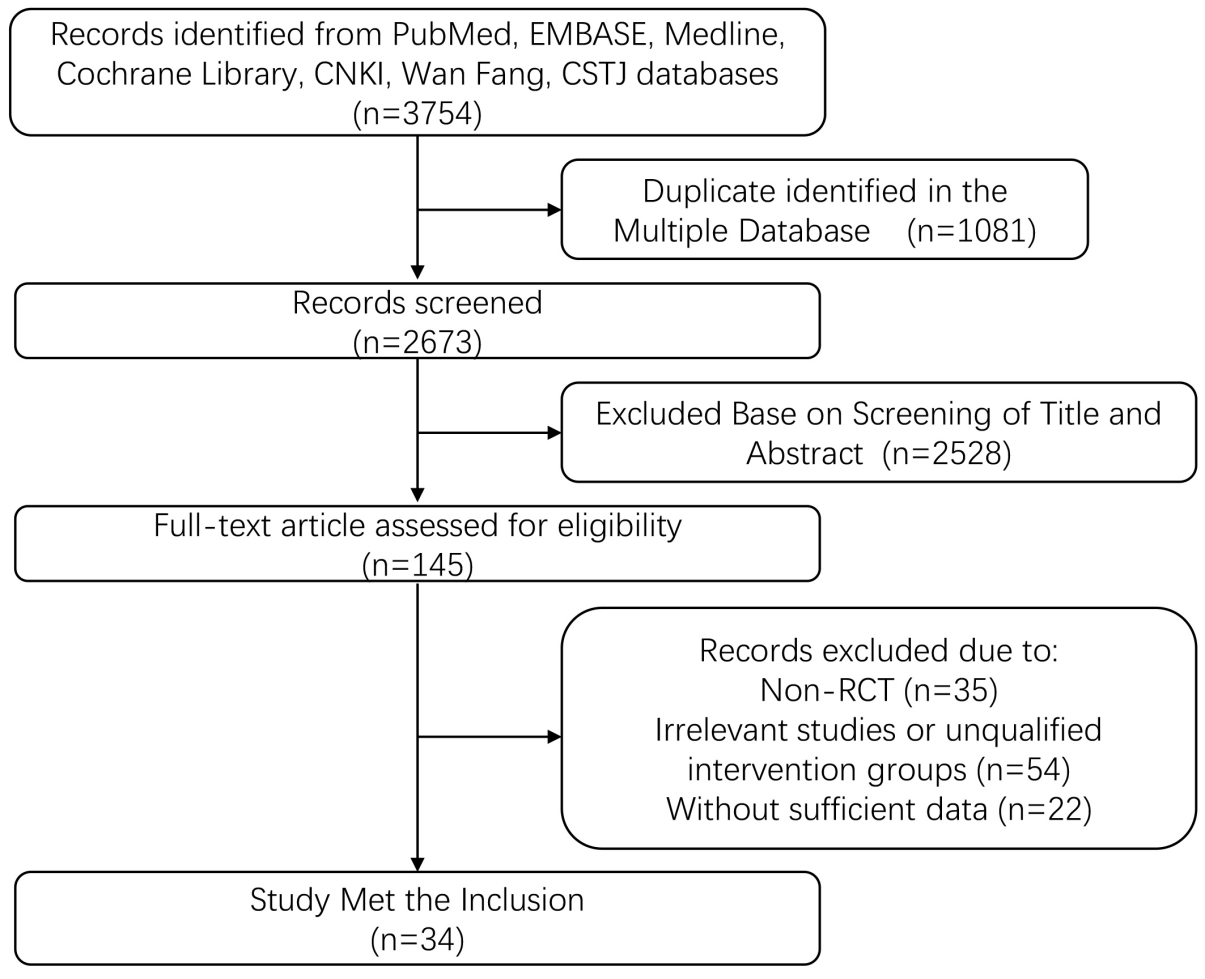
**Flow diagram showing the study selection process**. RCT, randomized controlled trial.

We searched the PubMed, Embase, Medline, Cochrane Library, CNKI, Wanfang, and CSTJ databases up to October 2022 to evaluate the efficacy of different types of combinations of antihypertensive drugs in controlling BP in hypertensive patients by using the following search terms: (a) hypertension and (b) valsartan. We identified gray literature by retrieving relevant institutions and clinical trial registries. All analyses were based on previously published studies and therefore did not require ethical approval or patient consent. The detailed search strategies are displayed in Fig. 1.

### 2.2 Eligibility Criteria

#### Inclusion and Exclusion Criteria

Inclusion criteria: (1) patients who were enrolled RCTs were diagnosed with essential hypertension; (2) studies compared different two-drug combination therapies of various doses (i.e., 80, 160, and 320 mg) of valsartan with each other or traditional therapies including valsartan; (3) studies reported changes in systolic blood pressure (SBP) and diastolic blood pressure (DBP) as well as adverse effects; and (4) full text was available for access.

Exclusion criteria: (1) non-RCT (i.e., narrative reviews and cohort studies); (2) unqualified intervention groups (e.g., combination or monotherapy studies without valsartan); (3) duplicate reports; (4) unable to extract sufficient, relevant data (no data of changes in SBP/DBP or adverse effects).

### 2.3 Data Extraction

All literature was imported into EndNote X9.3.3 software, Thomson ResearchSoft (Philadelphia, PA, USA) for screening and management. After removing duplicate studies, two reviewers independently screened the title and abstract of each study to judge the eligibility of the study. If the abstract and the title could not be used to determine the eligibility, the full text was downloaded for further evaluation. Disagreements between the reviewers were resolved by discussion or by consulting a third party. The following data were extracted objectively and faithfully with respect to the original data: study design, intervention methods, sample sizes, age, baseline disease (diabetes), baseline SBP and DBP, and outcome data (adverse effects, changes in SBP and DBP). Adverse effects in different studies were reported differently because of the treatment of different combination drugs. Therefore, permanent discontinuations related to treatment were the most accurate and objective measure of adverse effects. The common adverse effects of most studies (i.e., dizziness, headache, nasopharyngitis, asthenia and urticaria) were also included.

### 2.4 Statistical Analysis

ADDIS 1.16.7, drugis.org (Groningen, Groningen, NL, USA) and STATA 16, StataCorp LLC (College Station, TX, USA) were used for Bayesian model network meta-calculation. We used Markov chain Monte Carlo methods to perform 20,000 tuning iterations and 50,000 simulation iterations with 4 Markov chains. Based on the results of the orbit diagrams and density diagram, the degree of convergence of the model was determined. Continuous variables were analyzed using odds ratios (ORs) with 95% confidence intervals (CIs), with OR values less than 0 and 95% CI values less than 0 indicating a statistically significant difference. We use a node-splitting model to check that the trial analysis across the network is indeed consistent. In addition, when the 95% CI for the median discordance factor was zero, discordance was considered inconsequential if the discordance standard deviation was less than or equal to the random effects standard deviation. Probability values were summarized and reported as the surface under the cumulative ranking (SUCRA) curve. When a treatment is certain to be the worst, the SUCRA value is 0, and when it is certain to be the best, the SUCRA value is 1.

## 3. Results 

### 3.1 Study Characteristics

**Fig. 2.**
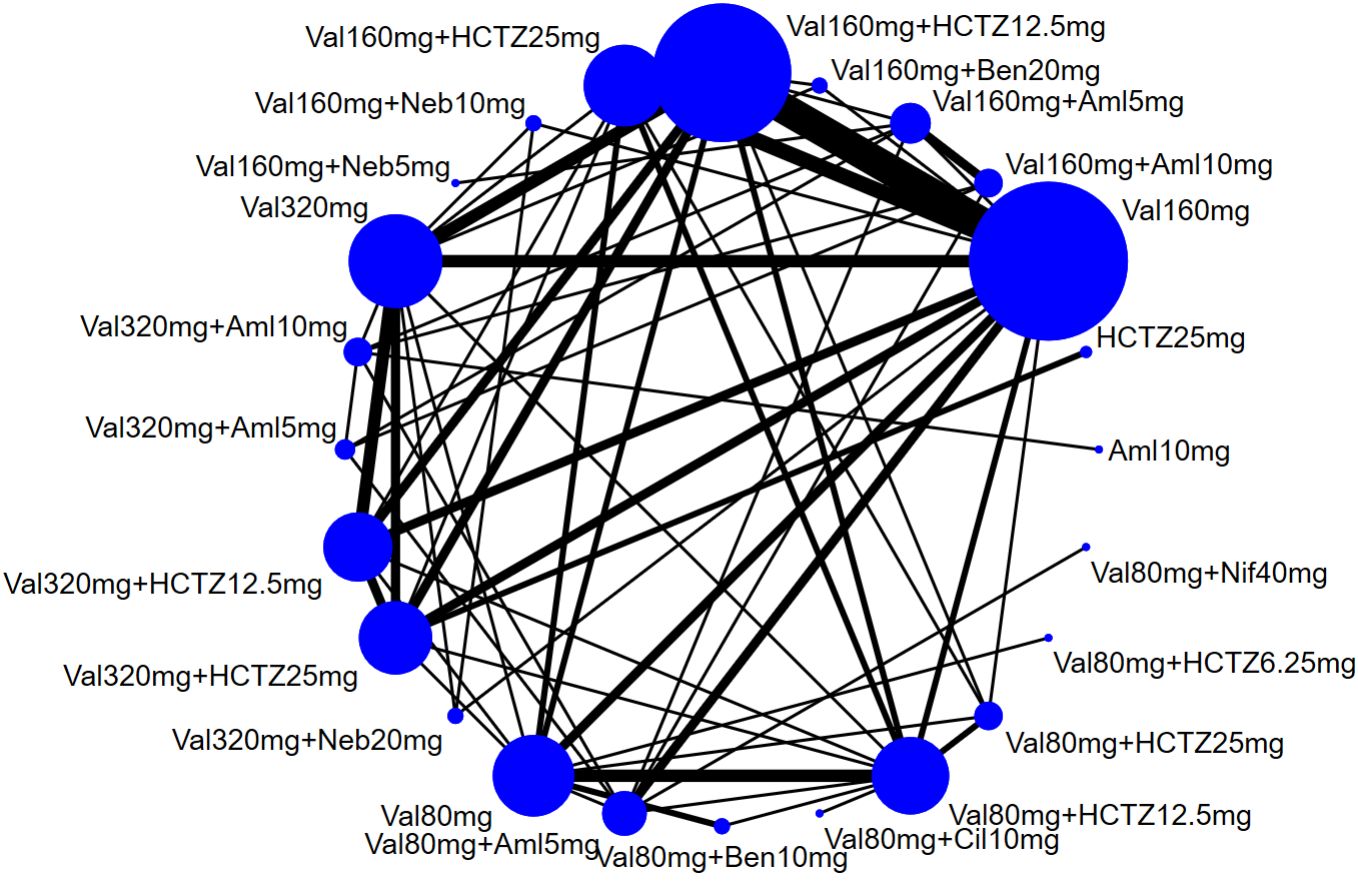
**The construction of the network**. Abbreviations: Val, valsartan; Aml, amlodipine; HCTZ, hydrochlorothiazide; Neb, nebivolol; Ben, benazepril; Nif, nifedipine; Cil, cilnidipine.

Overall, the systematic review and network meta-analysis included 34 clinical studies involving 26,752 hypertensive patients (**Supplementary Material**). In these RCTs, patients are randomly assigned to groups. The characteristics of the included studies and relevant patient characteristics are summarized in **Supplementary Table 1**. The outcomes of the included studies are summarized in **Supplementary Table 2**. The network comparison between different processing strategies is constructed as shown in Fig. 2.

### 3.2 Bayesian Network Meta-Analyses 

#### 3.2.1 Two-Drug Combinations Therapy of Valsartan with the Best Antihypertensive Effect on the Basis of Relatively Sufficient Blocking of ${\mathrm{AT}}_{1}$R

The results of the network meta-analysis showed that a relatively large dose of valsartan (320 mg) combined with amlodipine 10 mg had the best antihypertensive effect on SBP (compared with amlodipine 5 mg (mean: –7.14, –12.28 to –2.13), hydrochlorothiazide 12.5 mg (mean: –4.85, –10.39 to 0.67), hydrochlorothiazide 25 mg (mean: –3.76, –9.06 to 1.44), nebivolol 20 mg (mean: –10.23, –16.94 to –3.75)) and DBP (compared with amlodipine 5 mg (mean: –7.51, –10.27 to –4.78), hydrochlorothiazide 12.5 mg (mean: –5.94, –9.10 to –2.93), hydrochlorothiazide 25 mg (mean: –4.39, –7.53 to –1.49), nebivolol 20 mg (mean: –4.69, –8.44 to –1.14)) (**Supplementary Fig. 1**). It could also be seen from the SUCRA curve that valsartan 320 mg combined with amlodipine 10 mg had the best hypotensive effect (**Supplementary Figs. 2,3**). For SBP, valsartan 320 mg combined with hydrochlorothiazide 12.5 mg or hydrochlorothiazide 25 mg could also be used equivalently.

#### 3.2.2 Two-Drug Combinations Therapy of Valsartan for Relatively Sufficient Blocking of ${\mathrm{AT}}_{1}$R on the Basis of BP Reaching the Standard

Through observation of several two-drug combinations, it was found that when the dose of valsartan was increased from 80 mg to 320 mg, valsartan combined with hydrochlorothiazide 25 mg would not further reduce SBP (compared with valsartan 80 mg (mean difference: –2.00, –15.83 to 12.31), valsartan 160 mg (mean difference: –1.67, –4.92 to 1.59)) and DBP (compared with valsartan 80 mg (mean difference: –3.95, –8.71 to 0.66), valsartan 160 mg (mean difference: –0.85, –2.48 to 0.76)) significantly (**Supplementary Figs. 4–7**).

#### 3.2.3 Comparison of Adverse Effects of Different Two-Drug Combinations Therapy of Valsartan on the Basis of Relatively Sufficient Blocking of ${\mathrm{AT}}_{1}$R

There was no statistically significant difference in the adverse effects of valsartan 320 mg combination therapies (**Supplementary Fig. 8**). Compared with valsartan 80 mg combined with hydrochlorothiazide 25 mg and valsartan 160 mg combined with hydrochlorothiazide 25 mg, there was no significant increase in the above adverse effects of valsartan 320 mg combined with hydrochlorothiazide 25 mg (**Supplementary Fig. 9**). Additionally, the incidence of the above adverse effects would not increase compared with valsartan 320 mg alone.

## 4. Discussion

According to the latest statistics, the number of people with hypertension in China has reached 245 million. Residents over the age of 18 suffering from hypertension accounted for 27.9%, which means that 3 out of every 10 adults in China suffer from hypertension [6]. ARBs are the guideline-recommended first-line treatment for hypertension [12]. ARB binding to ${\mathrm{AT}}_{1}$R restrains the effects of Ang II, a member of the RAS.

Valsartan, similar to all ARBs, acts by inhibiting the binding of Ang II to ${\mathrm{AT}}_{1}$R to lower BP. Valsartan is the most commonly used ARB in China and many other countries [13]. Valsartan can effectively control BP to meet the requirements, but will not cause hypotension due to excessive BP reduction [14]. The initial dose of valsartan, 80 mg, shows comparable efficacy to some other ARBs (e.g., candesartan (8–16 mg), losartan (50–100 mg), irbesartan (150 mg), olmesartan (10 mg), and telmisartan (40 mg)) in patients with essential hypertension [13]. Moreover, valsartan administered at 160 or 320 mg is more effective at lowering BP than losartan 100 mg, irbesartan 150 mg and candesartan 16 mg [15]. Valsartan has good tolerability with a side-effect profile indistinguishable from placebo and superior to some other ARBs (e.g., olmesartan and losartan), which improved patient compliance, resulting in increased drug efficacy [16, 17, 18, 19]. One study showed total discontinuations in olmesartan, losartan and valsartan during treatment of 16.9%, 13.5% and 10.3%, respectively [20]. Additionally, there were more indications observed outside of hypertension of valsartan than some other ARBs, such as CVD, heart failure, kidney damage, etc. [14, 21, 22]. These advantages, in addition to the comparative cost-effectiveness of valsartan, indicate that valsartan remains a favorable option for ARB and combined treatment of hypertension [23, 24].

According to the results of the network meta-analysis, among many combination therapies, the combination of valsartan 320 mg with amlodipine 10 mg could better reduce BP without further adverse effects. Amlodipine, similar to other CCBs, acts primarily by inhibiting extracellular calcium influx through cardiac and vascular smooth muscle cell membranes [25]. Its main site of action is the peripheral vasculature, which is related to its direct relaxant effect on vascular smooth muscle, leading to dilation of both arteries and arterioles [25, 26]. A relatively large dose of valsartan could block ${\mathrm{AT}}_{1}$R more sufficiently, which combined with CCB with a complementary mechanism could better reduce BP [27]. In addition, for SBP, hydrochlorothiazide 12.5 mg or 25 mg is also equally recommended when the uric acid level is not high.

Additionally, the results of the network meta-analysis showed that when valsartan was combined with hydrochlorothiazide 25 mg, the increase in valsartan from 80 mg to 320 mg did not induce a further reduction in BP. With a single dose of valsartan blocking ${\mathrm{AT}}_{1}$R to relatively sufficient blocking ${\mathrm{AT}}_{1}$R, the above combined treatment would not affect BP but downregulate the expression of the angiotensin converting enzyme (ACE)-Ang II-${\mathrm{AT}}_{1}$ axis and upregulate the expression of the ACE2-angiotensin 1-7 (Ang (1-7))-MAS axis simultaneously [28, 29]. On the one hand, circulating Ang II levels tend to further increase and will be more combined with angiotensin type 2 receptor (${\mathrm{AT}}_{2}$R) [30, 31], which plays a role in anti-inflammation, antioxidation, reducing cardiomyocyte hypertrophy, anti-fibroblast proliferation and other related protective effects [31, 32]. On the other hand, the level of ACE2 is upregulated through feedback, which could convert Ang II to Ang (1-7) and then bind to the MAS receptor [33]. It can also improve oxidative stress, cell proliferation, inflammation, etc. [34]. The above mechanism could highlight the fact that a relatively large dose of valsartan combined with other treatments induces improvements in clinical complications [35, 36, 37, 38, 39].

The network meta-analysis results also showed that combined treatment with a relatively large dose of valsartan did not increase the incidence of permanent discontinuations related to treatment. The occurrence of other adverse effects was not higher than that of low-dose valsartan. The reduction in adverse effects of the combination of valsartan with hydrochlorothiazide or amlodipine may be attributed to the complementary mode of action by acting through different pathophysiologic pathways to offset each drug’s side effects [11].

## 5. Limitations

The first limitation of this study is that the classification of the included population is not detailed enough. The included studies did not compare patients of different sexes. Therefore, the study could further analyze whether there were differences in changes in BP in patients of different sexes after treatment. Second, some of the articles we included did not mention detailed quantitative data on adverse effects. We can only rely on the statements in the article as evidence. Third, some patient baseline disease and hypertension course time information was incomplete, so no analysis was conducted. Fourth, valsartan 320 mg combined with amlodipine 5 mg could not further reduce BP as well. However, due to the limited number of included studies and samples on this combined treatment, it may lead to deviation of the results. Studies with large sample sizes or randomized controlled clinical trials should be conducted in real-world settings to further validate these results. Fifth, for the combination of two drugs, it was not discussed whether it was a single drug combination or compound preparation.

## 6. Conclusions

In conclusion, combination therapy with a relatively large dose of valsartan could control BP and improve clinical complications effectively. However, for hypertensive patients with different treatment requirements, we should make specific choices about whether to control BP, improve clinical complications, or both.

## Data Availability

The datasets used or analysed during the study are available from the corresponding author on reasonable request.

## Supplementary Material

Supplementary material associated with this article can be found, in the online version, at https://doi.org/10.31083/j.rcm2407187.
