# Supplementary material for: Two-Drug Combinations Therapy of Different Doses of Valsartan Existing Diverse Significance for Hypertensive Patients
Source: Rev Cardiovasc Med. 2023 Jun 29;24(7):187. doi: 10.31083/j.rcm2407187 (PMC11266496; doi:10.31083/j.rcm2407187)
Supplement: Supplementary file 1 [file 2153-8174-24-7-187-s1.zip › Supplementary Material.docx]

**Supplementary Table 1. Characteristics of the studies included in this meta-analysis**

| **Study (author, year)** | **Interventions** | **Sample size** | **Mean age (year)** | **Diabetes n (%)** | **Baseline SBP (mmHg)** | **Baseline DBP (mmHg)** |
| --- | --- | --- | --- | --- | --- | --- |
| Kang-Ling Wang 2020  [1] | Val80mg + Aml5mg/Val 160mg | 21/21 | 59.5 ± 13.8/55.1 ± 11.8 | 3(14.3)/4(19.0) | 150.3 ± 12.9/141.1 ± 12.7 | 92.1 ± 10.8/90.0 ± 9.4 |
| Bernard Waeber 2001 [2] | Val 80mg/Val80mg + Benazepril10mg/Val80mg + HCTZ12.5mg | 179/74/74 | 56 ± 12/56 ± 11/57 ± 10 | n.a | 159.1 ± 16.7/163.0 ± 15.0/163.5 ± 15.4 | 101.0 ± 5.8/102.3 ± 4.3/102.9 ± 5.5 |
| S ZHANG 2008 [3] | Val80mg + HCTZ12.5mg/Val80mg | 61/62 | 54.82 ± 8.04/54.40 ± 8.38 | n.a | 150.56 ± 11.42/148.23 ± 12.43 | 98.64 ± 4.73 /98.31 ± 5.29 |
| David T. Nash 2015 [4] | Val160mg/Val160mg + HCTZ12.5mg | 263/261 | 52.2 ± 10.9/53.0 ± 10.6 | n.a | 151.0 ± 13.9/151.6 ± 13.5 | 99.9 ± 4.0/99.8 ± 4.0 |
| Daniel A. Duprez 2011 [5] | Val160mg+HCTZ12.5mg/ Val160mg | 33/41 | 77.0 ± 3.7/ 78.2 ± 3.9 | 5(15.2)/6(14.6) | 161.3 ± 14.5/ 163.3 ± 8.7 | 83.5 ± 10.2/ 85.1 ± 9.5 |
| Yves Allemann 2008 [6] | Val160mg + Aml5mg/Val160mg + Aml10mg | 440/449 | 58.5 ± 12.3/58.6 ± 12.0 | 72(16.3)/72(16.3) | 149.8 ± 9.7/ 150.4 ± 10.5 | 90.8 ± 7.7/ 90.6 ± 7.6 |
| T. R. Smith 2010 [7] | Val160mg + Aml5mg/Val160mg + Aml10mg | 605/605 | 53.9 ± 11.0/54.5 ± 11.5 |  | 152.1 ± 12.9/151.7 ± 12.4 | 99.0 ± 3.4/ 98.8 ± 3.2 |
| Dingliang Zhu 2013 [8] | Val160mg + Aml5mg/Val160mg | 328/323 | 52.9 ± 9.47/53.1 ± 9.41 | 31(9.5)/40(12.4) | 146.9 ± 11.71/146.3 ± 12.81 | 96.7 ± 5.15/96.2 ± 4.86 |
| Luis M. Ruilope 2005 [9] | Val160mg+HCTZ12.5mg/ Val160mg+HCTZ25mg | 357/363 | 61±11/ 61±11 | 64(17.9)/61(16.8) | 167±5/ 166±5 | 94±8/ 93±8 |
| Yves Lacourci~re 2005 [10] | Val160mg + HCTZ25mg/Val160mg + HCTZ12.5mg/Val160mg | 252/254/261 | 60.7 ± 11.6/60.8 ± 11.5/  60.4 ± 10.6 | n.a | 167.2 ± 7.9/167.4 ± 8.3/167.9 ± 8.0 | 93.7 ± 8.8/93.4 ± 9.6/93.2 ± 8.9 |
| JEAN-MICHEL MALLION 2003 [11] | Val160mg/Val160mg + HCTZ12.5mg/  Val160mg + HCTZ25mg | 663/665/666 | 55.3 ± 11.2/56.0 ± 11.1/55.7 ± 11.2 | n.a | 160.2 ± 12.40/160.5 ± 12.68/160.4 ± 12.22 | 101.3 ± 4.06/101.4 ± 4.11/101.5 ± 3.99 |
| Matthew R. Weir 2001 [12] | Val160mg + Benazepril20mg/Val160mg + HCTZ12.5mg/Val320mg | 30/30/28 | 45 ± 8/47 ± 10/47 ± 6 | n.a | 149 ± 16/144 ± 21/145 ± 16 | 101 ± 10/96 ± 11/95 ± 9 |
| Shigeru Toyoda 2015 [13] | Val80mg + HCTZ12.5mg/ Val80mg + Aml5mg | 38/49 | 67 ± 13/ 62 ± 13 | n.a | 156 ± 17/162 ± 19 | 86 ± 12/95 ± 13 |
| Ikuo SAITO 2006 [14] | Val80mg + Nfedipine40mg/Val80mg + Aml5mg | 245/260 | 57.5 ± 10.7/56.3 ± 11.0 | 6(2.4)/13(5.0) | 161.9 ± 12.7/161.6 ± 13.1 | 100.4 ± 9.1 /102.3 ± 8.0 |
| Dion Zappe 2009 [15] | Val160mg/Val160mg + HCTZ12.5mg/Val160mg + HCTZ25mg/ Val320mg + HCTZ25mg | 641 | 54.6 ± 10.8 | n.a | 150.4 ± 9.0 | 93.9 ± 6.4 |
| Julián Segura 2003 [16] | Val160mg/ Val160mg + Benazepril20mg | 12/12 | 49.7 + 12.4/47.9 + 15.2 | n.a | 152 + 21/149 + 15 | 89 + 10/87 + 10 |
| Gokhan Alici 2008 [17] | Val80mg/ Val80mg + HCTZ6.25mg | 20/20 | 53.00 ± 12.63/55.25 ± 9.04 | 4(20.0)/7(35.0) | 159.9 ± 8.4/ 155.6 ± 6.9 | 95.4 ± 8.3/ 91.7 ± 4.3 |
| Jorge Sison 2014 [18] | Val80mg + Aml5mg/Val160mg + Aml5mg/Val160mg + Aml10mg/Val320mg + Aml5mg/Val320mg +Aml10mg | 1428/4839/  1941/28/24 | 52.8 ± 11.32/56.0 ± 11.15 | 2663 (31.0) | 155.0/160.4/166.5/  161.8/169.1 | 92.6/97.5/99.4/  96.4/103.3 |
| Jun Huang 2011 [19] | Val80mg + Aml5mg/ Val80mg/ Val160mg | 308/306/302 | 51.6 ± 10.8/51.7 ± 8.9/51.2 ± 9.6 | 16(5.2)/23(7.5)/13(4.3) | 141.7 ± 12.7/141.9 ± 13.1/139.4 ± 12.5 | 95.1 ± 4.7/95.5 ± 4.8/95.1 ± 4.8 |
| Takeshi Fujiwara 2020 [20] | Val80mg + cilnidipine10mg/Val80mg + HCTZ12.5mg | 63/66 | n.a | n.a | 143.9 ± 17.7/ 144.2 ± 18.1 | 82.9 ± 13.3/ 85.2 ± 11.6 |
| Elizabeth O. Olifi 2011 [21] | White: Val320mg+Aml10mg/Val160mg+Aml5mg  African American: Val320mg+Aml10mg/Val160mg+Aml5mg  Hispanic: Val320mg+Aml10mg/Val160mg+Aml5mg | 235/239/105/93/84/81 | 55.7±11.5/56.4±11.1/50.8±9.3/51.8±10.4/53.5±11.4/56.5±11.1 | 49(20.5)/34(14.5)/9(9.7)/11(10.5)/19(23.5)/14(16.7) | 163.0±11.3/163.4±11.2/165.4±12.9/163.7±12.1/162.0±10.0/164.2±11.5 | 94.3±11.1/94.4±10.9/98.4±11.6/96.8±9.2/92.5±10.7/91.1±10.1 |
| James L. Pool 2007 [22] | Val160mg/Val320mg/Val160mg + HCTZ12.5mg/Val320mg + HCTZ12.5mg/Val320mg + HCTZ25mg | 166/170/  168/168/  169 | 52.2 ± 11.2/52.5 ± 11.8/  52.9 ± 10.4/52.1 ± 11.4/  53.5 ± 11.1 | n.a | 149.6 ± 13.0/149.5 ± 13.1/  150.0 ± 12.0/150.7 ± 12.7/  152.5 ± 11.7 | 98.9 ± 3.2/99.0 ± 4.0/  99.1 ± 3.6/99.2 ± 3.7/  99.4 ± 3.9 |
| Thomas D Giles 2014 [23] | Val160mg + Nebivolol10mg/Val320mg + Nebivolol20mg/Val160mg/ Val320mg | 555/554/555/554 | 50.9 ± 10.1/50.8 ± 9.7/  51.7 ± 9.9/51.1 ± 10.7 | 81(15)/89(16)/84(15)/88(16) | 154.6 ± 11.8/154.6 ± 11.5/  155.8 ± 12.1/155.1 ± 11.7 | 99.6 ± 3.5/99.9 ± 3.7/  99.8 ± 3.8/99.7 ± 3.6 |
| JR Benz 1998 [24] | Val80mg/Val160mg/Val80mg + HCTZ12.5mg/  Val160mg + HCTZ12.5mg/Val80mg + HCTZ25mg/  Val160mg + HCTZ25mg | 99/97/96/96/92/94 | 52 ± 10.2/52 ± 10.5/52 ± 11.9/53 ± 11.3/51 ± 11.2/53 ± 11.2 | n.a | 153.7 ± 14.4/153.5 ± 15.1/153.0 ± 14.4/154.5 ± 15.4/152.0 ± 14.2/155.9 ± 14.8 | 101.5 ± 4.9/101.5 ± 4.8/101.0 ± 4.9/101.0 ± 4.5/100.4 ± 4.6/101.4 ± 4.8 |
| Rufeng Shi 2016 [25] | Val80mg + Aml5mg/Val160mg | 38/41 | n.a | n.a | n.a | n.a |
| Selvia M. Farag 2018 [26] | Val160mg + Aml10mg/ Val160mg + Nebivolol5mg | 75/62 | 55.44 ± 11.15/57.5 2± 10.24 | 19(25.7)/18(30.5) | n.a | n.a |
| YoungKeun Ahn2018 [27] | Val160mg + Aml5mg/ Val160mg + HCTZ12.5mg | 121/117 | 56.14 ± 9.97/57.74 ± 11.29 | n.a | 144.58 ± 10.62/148.43 ± 12.34 | 96.26 ± 5.43/97.66 ± 6.92 |
| Brendan M. Everett 2008 [28] | Val320mg/Val320mg + HCTZ12.5mg | 836/832 | 50 ± 3.83/50 ± 4.08 | 97(11.6)/69(8.3) | 164.0 ± 13.0/165.0 ± 13.0 | 100.0 ± 9.0/100.0 ± 9.0 |
| DH Zappe 2010 [29] | Val160mg/Val160mg + HCTZ12.5mg/  Val160mg + HCTZ25mg | 218/221/213 | 52.2 ± 10.7/53.1 ± 9/ 52.6 ± 10.4 | 23(10.6)/30(13.6)/30(14.1) | 160.7 ± 7.4/161.1 ± 7.9/161.0 ± 7.8 | 97.7 ± 5.5/98.2 ± 5.1/  98.2 ± 5.1 |
| Elizabeth O. Ofili 2013 [30] | African Americans:Val320mg + HCTZ25mg/HCTZ25mg  Whites:Val320mg + HCTZ25mg/HCTZ25mg | 67/59/103/109 | African Americans:52.7 ± 7.2/53.4 ± 8.8  Whites:58.7 ± 9.2/57.2 ± 8.1 | 1(1)/5(8)/4(4)/6(6) | African Americans:159.6 ± 8.4/159.6 ± 7.8  Whites:159.9 ± 7.8/159.7 ± 7.9 | African Americans:96.1 ± 6.5/94.6 ± 8.8  Whites:94.6 ± 8.2/93.3 ± 8.2 |
| Helmut Geiger 2009 [31] | HCTZ25mg/Val320mg + HCTZ25mg | 151/164 | 52.6 ± 9.93/55.0 ± 11.40 | 21(13.8)/19(12.3) | 154.1 ± 12.61/156.7 ± 12.49 | 99.9 ± 4.33/99.9 ± 3.97 |
| JM Flack 2009 [32] | Val320mg + Aml10mg/Aml10mg | 286/285 | 52.9/53.6 | 47(16.4)/41(14.3) | 170.4 ± 9.6/170.5 ± 8.9 | 98.5 ± 10.9/98.2 ± 10.1 |
| STEVEN G. CHRYSANT  1998 [33] | Val80mg + HCTZ12.5mg/Val80mg + HCTZ25mg | 18/55 | 52.9 ± 10.2/55.3 ± 10.4 | n.a | 156.7 ± 19.5/153.4 ± 14.7 | 99.7 ± 3.5/99.8 ± 3.8 |
| Yu Bo 2011 [34] | Aml10mg/Val80mg + Aml5mg | 100/100 | 53.8 ± 7.6 | n.a | 149.1 ± 11/152.8 ± 11.3 | 97.7 ± 3.0/ 98.1 ± 3.3 |

Abbreviations: Val valsartan, Aml amlodipine, HCTZ hydrochlorothiazide, Neb nebivolol, Ben benazepril, Nif nifedipine, Cil cilnidipine, n.a. not available.

**Supplementary Table 2. Outcomes of the studies included in this meta-analysis**

| **Study (author, year)** | **Interventions** | **Changes in SBP (mmHg)** | **Changes In DBP (mmHg)** | **Permanent**  **discontinuations (n)** |
| --- | --- | --- | --- | --- |
| Kang-Ling Wang 2020  [1] | Val80mg + Aml5mg/Val 160mg | -16.5±15.5/-6.9±11.4 | -9.8±7.7/-2.5±6.6 | 0/4 |
| Bernard Waeber 2001 [2] | Val 80mg/Val80mg + Benazepril10mg/Val80mg + HCTZ12.5mg | -0.6±11.3/-3.2±12.1/-6.4±15.4 | -0.5±6.9/-3.3±8.0/-4.5±8.0 | 8/1/4 |
| S ZHANG 2008 [3] | Val80mg + HCTZ12.5mg/Val80mg | -15.77±13.17/-9.53±13.55 | -11.74±6.66/-9.66±7.64 | n.a |
| David T. Nash 2015 [4] | Val160mg/Val160mg + HCTZ12.5mg | -10.9±1.05/-16.8±1.16 | -11.2±0.67/-14.2±0.83 | 8/10 |
| Daniel A. Duprez 2011 [5] | Val160mg+HCTZ12.5mg/ Val160mg | n.a | n.a | n.a |
| Yves Allemann 2008 [6] | Val160mg + Aml5mg/Val160mg + Aml10mg | -17.5±0.7/-20.0±0.7 | -10.4±0.4/-11.6±0.4 | 25/62 |
| T. R. Smith 2010 [7] | Val160mg + Aml5mg/Val160mg + Aml10mg | -21.7±13.6/-22.9±13.7 | -16.4±7.1/-17.1±7.1 | 17/44 |
| Dingliang Zhu 2013 [8] | Val160mg + Aml5mg/Val160mg | Older: -18.7±12.31/-12.8±10.38  Younger: -14.6±11.76/-6.4±12.62 | Older: -12.8±7.85/-8.9±7.47  Younger: -10.1±7.09/-6.3±8.0 | 5/4 |
| Luis M. Ruilope 2005 [9] | Val160mg+HCTZ12.5mg/ Val160mg+HCTZ25mg | -21.7±13.7/-29.7±13.7 | -9.5±7.7/-11.1±7.7 | 15/13 |
| Yves Lacourci~re 2005 [10] | Val160mg + HCTZ25mg/Val160mg + HCTZ12.5mg/Val160mg | -27.9±13.8/-28.3±13.1/-20.7±15.7 | -10.2±7.7/-10.1±7.8/-6.6±8.9 | 13/12/12 |
| JEAN-MICHEL MALLION 2003 [11] | Val160mg/Val160mg + HCTZ12.5mg/  Val160mg + HCTZ25mg | -15.7±13.32/-19.4±14.56/-21.8±13.91 | -10.3±8.43/-12.8±8.22/-14.2±7.77 | 10/12/16 |
| Matthew R. Weir 2001 [12] | Val160mg + Benazepril20mg/Val160mg + HCTZ12.5mg/Val320mg | 1.0±12.0/-10.0±20.0/-4.0±23.0 | -4.0±10.0/-6.0±14.0/-2.0±12.0 | n.a |
| Shigeru Toyoda 2015 [13] | Val80mg + HCTZ12.5mg/ Val80mg + Aml5mg | n.a | -7.0±9.0/-13.0±11.0 | n.a |
| Ikuo SAITO 2006 [14] | Val80mg + Nfedipine40mg/Val80mg + Aml5mg | -34.0±15.0/-27.0±14.5 | -20.1±9.5/-15.9±9.7 | 7/6 |
| Dion Zappe 2009 [15] | Val160mg/Val160mg + HCTZ12.5mg/Val160mg + HCTZ25mg/ Val320mg + HCTZ25mg | -15.3±0.5/-19.6±0.5/-21.4±0.5/-22.3±0.5 | -8.9±0.3/-10.6±0.4/-12.1±0.3/-12.8±0.3 | n.a |
| Julián Segura 2003 [16] | Val160mg/ Val160mg + Benazepril20mg | -15.0±8.0/-12.0±11.0 | -4.0±6.0/-5.0±8.0 | n.a |
| Gokhan Alici 2008 [17] | Val80mg/ Val80mg + HCTZ6.25mg | -19.5±2.79/-21.25±4.12 | -9.75±1.88/-10.3±1.52 | n.a |
| Jorge Sison 2014 [18] | Val80mg + Aml5mg/Val160mg + Aml5mg/Val160mg + Aml10mg/Val320mg + Aml5mg/Val320mg +Aml10mg | -26.1±0.48/-30.7±0.22/-35.5±0.43/-31.0±3.0/-39.2±3.3 | -13.7±0.29/-16.8±0.14/-18.2±0.25/-13.5±2.25/-21.0±2.1 | n.a |
| Jun Huang 2011 [19] | Val80mg + Aml5mg/ Val80mg/ Val160mg | n.a | n.a | 6/0/5 |
| Takeshi Fujiwara 2020 [20] | Val80mg + cilnidipine10mg/Val80mg + HCTZ12.5mg | -11.8±15.9/-1.8±16.4 | -5.6±10.4/-8.3±9.5 | n.a |
| Elizabeth O. Olifi 2011 [21] | White: Val320mg+Aml10mg/Val160mg+Aml5mg  African American: Val320mg+Aml10mg/Val160mg+Aml5mg  Hispanic: Val320mg+Aml10mg/Val160mg+Aml5mg | n.a | n.a | 9/19 |
| James L. Pool 2007 [22] | Val160mg/Val320mg/Val160mg + HCTZ12.5mg/Val320mg + HCTZ12.5mg/Val320mg + HCTZ25mg | -14.5±0.98/-13.7±0.98/-20.3±0.98/-21.7±0.97/-24.7±0.99 | -11.7±0.65/-11.3±0.65/-15.2±0.65/-15.0±0.64/-16.6±0.65 | n.a /13/7/5/5 |
| Thomas D Giles 2014 [23] | Val160mg + Nebivolol10mg/Val320mg + Nebivolol20mg/Val160mg/ Val320mg | -17.7±15.3/-17.8±15.8/-14.2±14.4/-14.8±15.1 | -14.8±9.2/-15.7±9.6/-10.8±9.6/-11.2±9.3 | 15/9/10/10 |
| JR Benz 1998 [24] | Val80mg/Val160mg/Val80mg + HCTZ12.5mg/  Val160mg + HCTZ12.5mg/Val80mg + HCTZ25mg/  Val160mg + HCTZ25mg | -6.89±2.28/-10.19±2.03/-14.59±2.02/-15.84±2.03/-19.23±2.06/-20.53±2.03 | -5.3±1.24/-5.3±1.23/-7.71±1.23/-9.39±1.23/-11.6±1.26±/-11.19±1.23 | 2/2/1/3/n.a/7 |
| Rufeng Shi 2016 [25] | Val80mg + Aml5mg/Val160mg | -20.24±7.35/-20.16±6.93 | -8.34±6.56/-8.67±6.59 | n.a |
| Selvia M. Farag 2018 [26] | Val160mg + Aml10mg/ Val160mg + Nebivolol5mg | -40.87±17.05/-34.31±11.32 | -16.31±11.77/-15.16±11.32 | n.a |
| YoungKeun Ahn2018 [27] | Val160mg + Aml5mg/ Val160mg + HCTZ12.5mg | -12.17±10.5/-11.24±1.08 | -9.44±0.69/-7.47±0.71 | n.a |
| Brendan M. Everett 2008 [28] | Val320mg/Val320mg + HCTZ12.5mg | -18.0±17.0/-25.0±18.0 | -10.0±10.0/-13.0±10.0 | 14/21 |
| DH Zappe 2010 [29] | Val160mg/Val160mg + HCTZ12.5mg/  Val160mg + HCTZ25mg | -15.3±12.0/-22.0±13.0/-26.0±14.0 | n.a | 6/5/6 |
| Elizabeth O. Ofili 2013 [30] | African Americans:Val320mg + HCTZ25mg/HCTZ25mg  Whites:Val320mg + HCTZ25mg/HCTZ25mg | African Americans:-15.0±4.6/-10.4±3.7  Whites:-19.1±2.2/-3.5±1.9 | African Americans:-9.7±3.6/-6.4±3.0  Whites-9.5±1.3/-1.3±1.1 | n.a |
| Helmut Geiger 2009 [31] | HCTZ25mg/Val320mg + HCTZ25mg | -6.0±1.12/-18.0±1.12 | -6.0±0.7/-14.0±0.7 | 4/5 |
| JM Flack 2009 [32] | Val320mg + Aml10mg/Aml10mg | -32.0±2.1/-24.5±2.1 | n.a | 7/9 |
| STEVEN G. CHRYSANT  1998 [33] | Val80mg + HCTZ12.5mg/Val80mg + HCTZ25mg | -11.7±25.9/-16.4±15.1 | -12.5±5.9/-12.6±6.8 | 2/3 |
| Yu Bo 2011 [34] | Aml10mg/Val80mg + Aml5mg | -21.5±11.1/-21.0±12.6 | -16.1±4.7/-16.0±6.6 | n.a |

Abbreviations: Val valsartan, Aml amlodipine, HCTZ hydrochlorothiazide, Neb nebivolol, Ben benazepril, Nif nifedipine, Cil cilnidipine, n.a. not available.


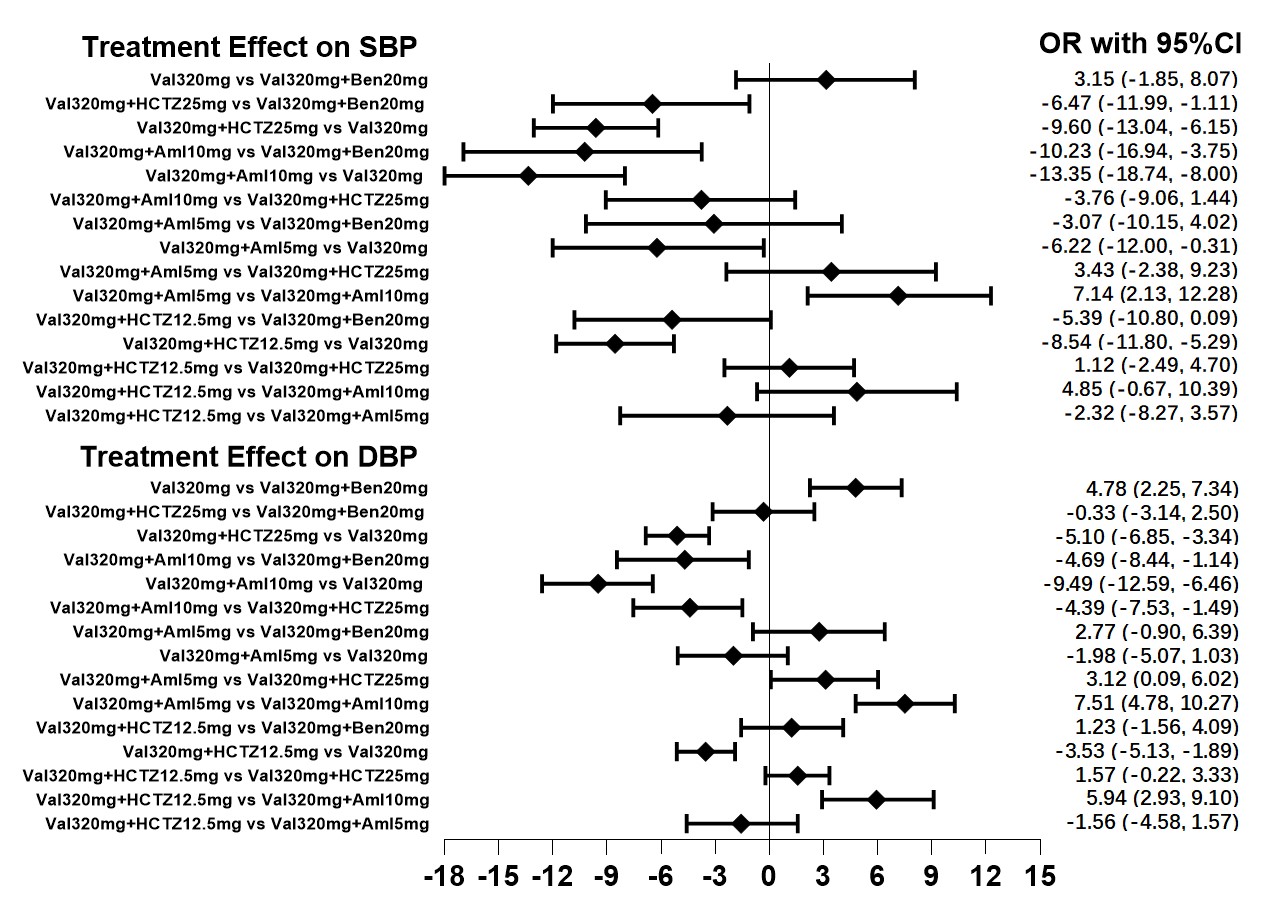


**Supplementary Fig. 1. Network forest plot of different two-drug combinations therapy of valsartan 320 mg on SBP and DBP.**


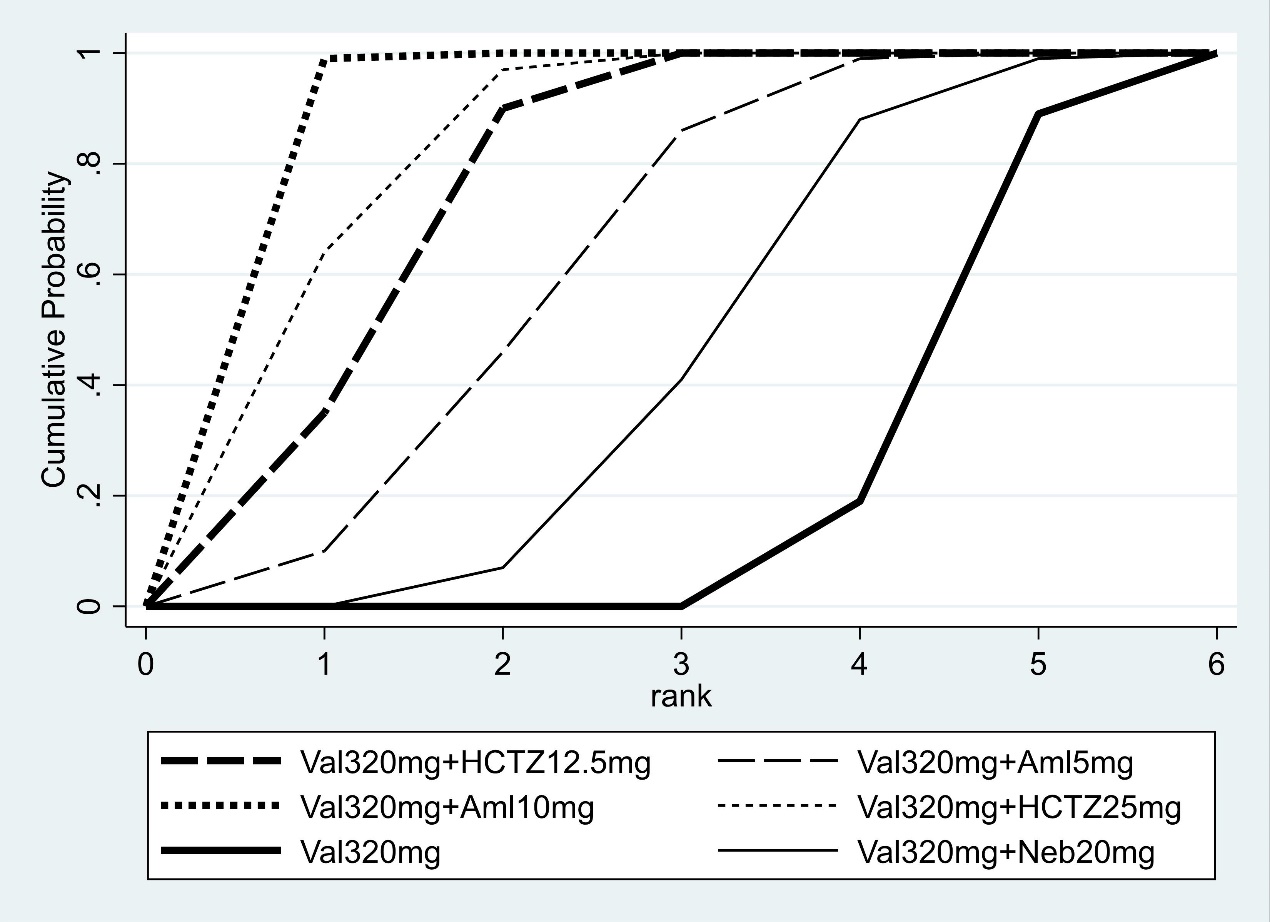


**Supplementary Fig. 2. The influence of 320 mg valsartan combined with different drugs on SBP.**


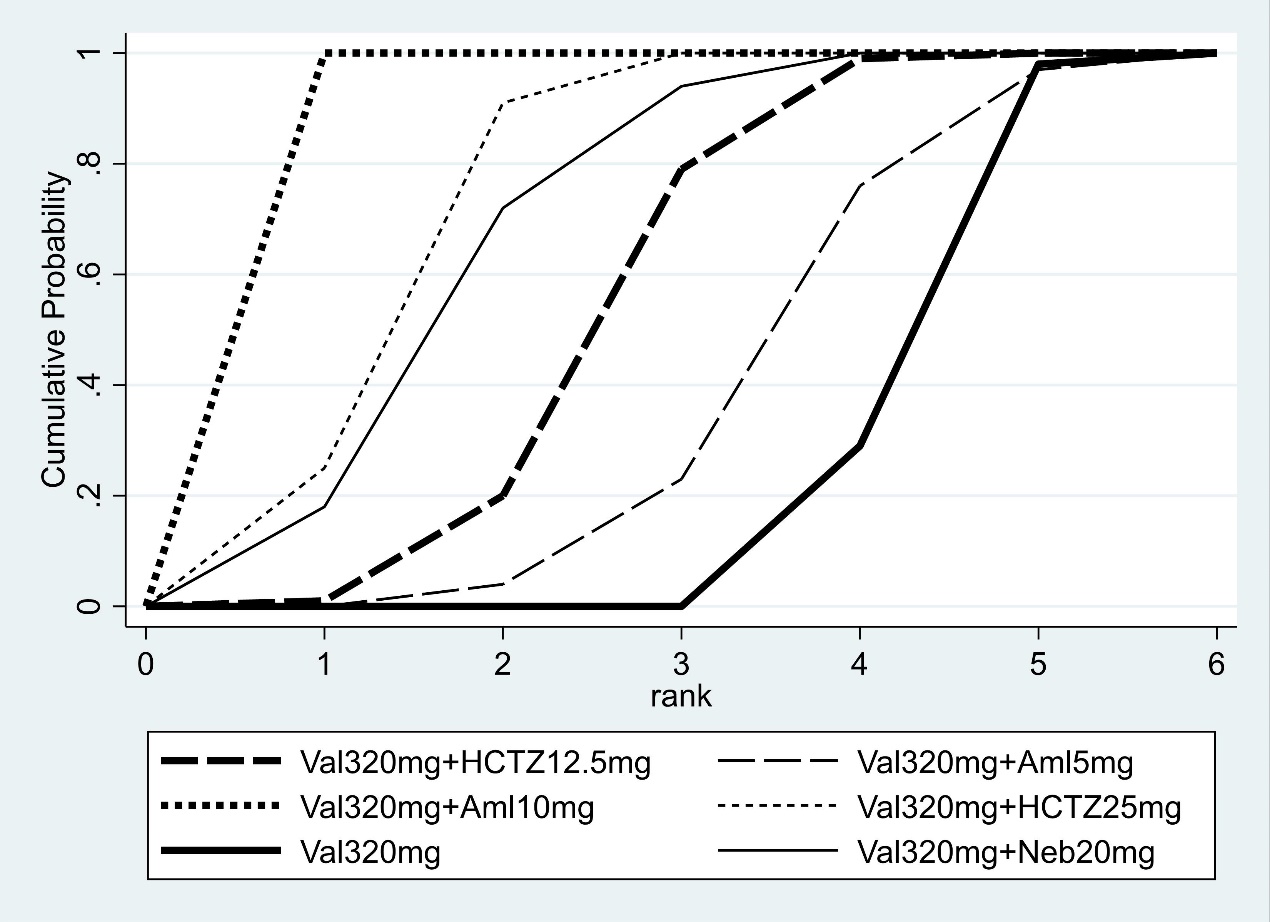


**Supplementary Fig. 3. The influence of 320 mg valsartan combined with different drugs on DBP.**


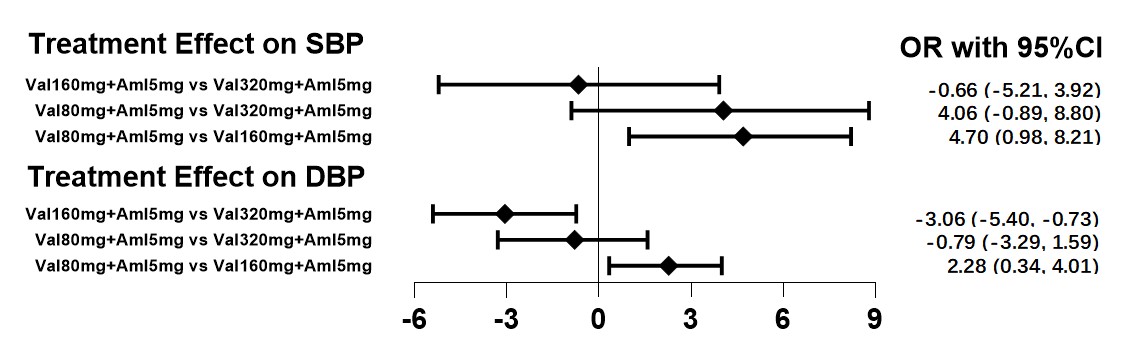


**Supplementary Fig. 4. Network forest plot of valsartan combined with amlodipine 5 mg on SBP and DBP.**


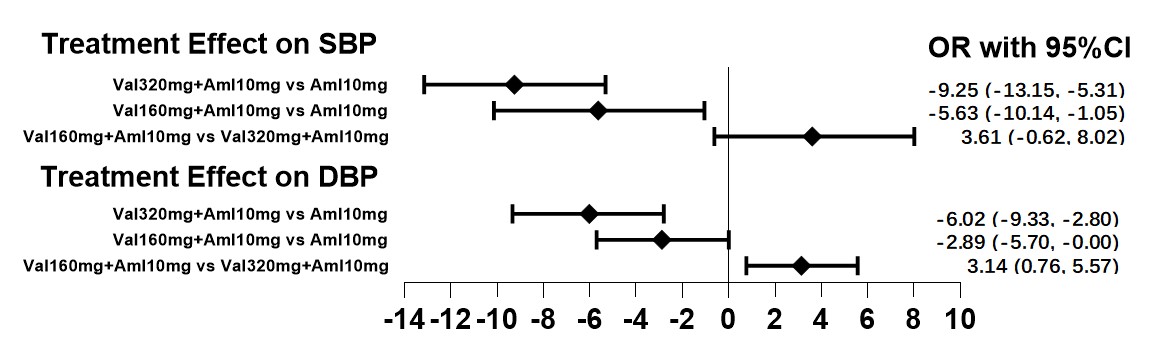


**Supplementary Fig. 5. Network forest plot of valsartan combined with amlodipine 10 mg on SBP and DBP.**


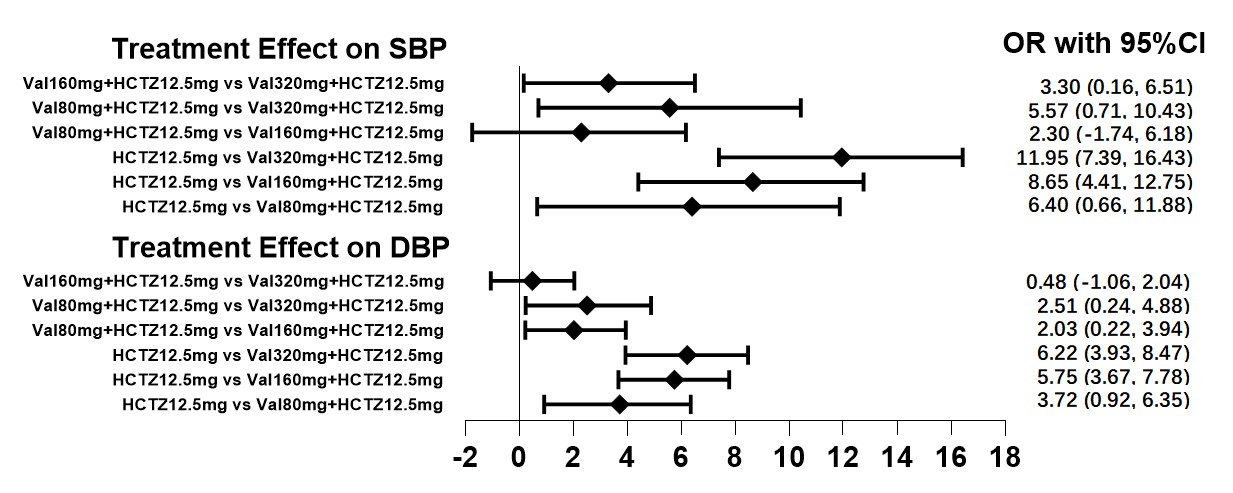


**Supplementary Fig. 6. Network forest plot of valsartan combined with hydrochlorothiazide 12.5 mg on SBP and DBP.**


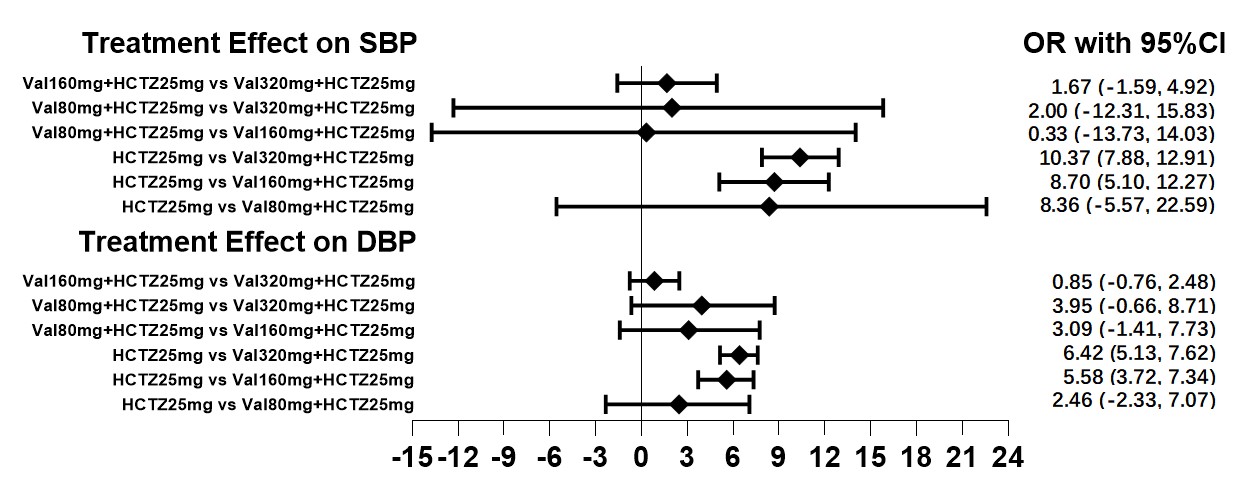


**Supplementary Fig. 7. Network forest plot of valsartan combined with hydrochlorothiazide 25 mg on SBP and DBP.**


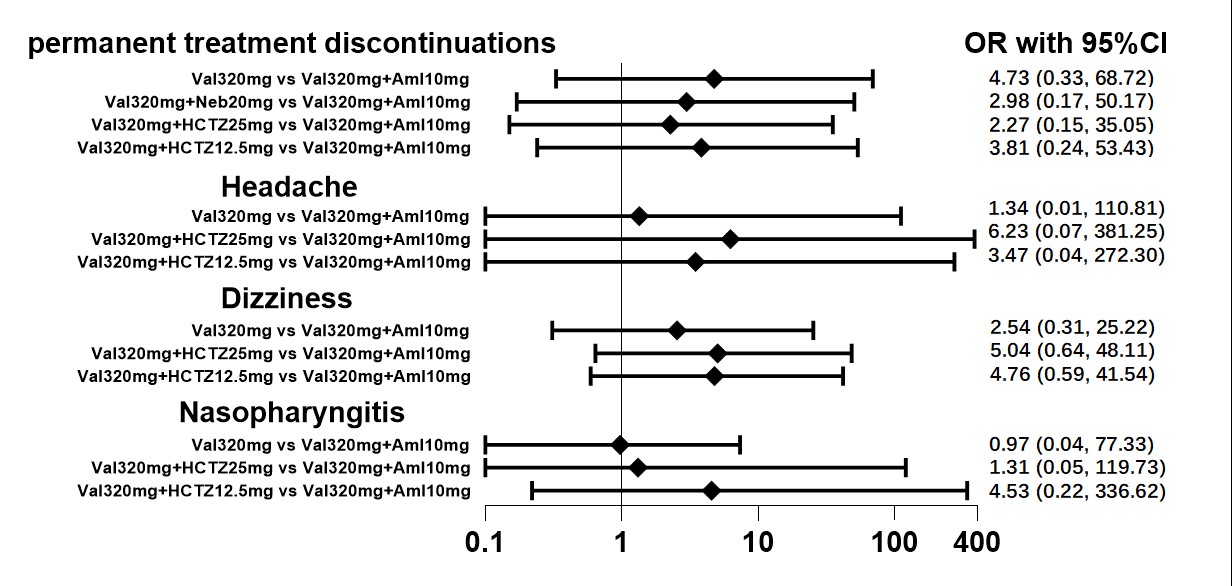


**Supplementary Fig. 8. Network forest plot of adverse effects of two-drug combinations therapy of valsartan with the best antihypertensive effect.**


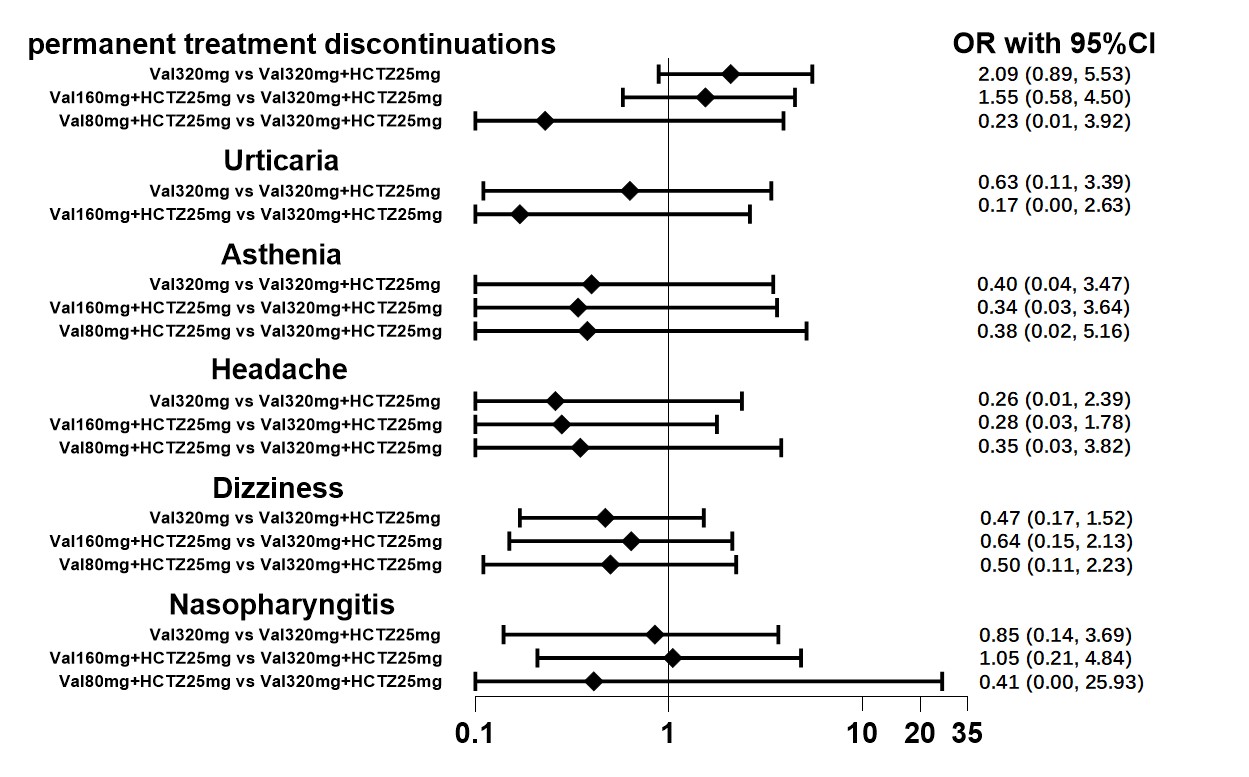


**Supplementary Fig. 9. Network forest plot of adverse reactions of two-drug combinations therapy of valsartan for relatively sufficient blocking of AT_1_R.**

References

[1] Wang KL, Yu WC, Lu TM, Chen LC, Leu HB, Chiang CE. Amlodipine/valsartan fixed-dose combination treatment in the management of hypertension: A double-blind, randomized trial. Journal of the Chinese Medical Association. 2020; 83: 900–905.

[2] Waeber B, Aschwanden R, Sadecky L, Ferber P. Combination of hydrochlorothiazide or benazepril with valsartan in hypertensive patients unresponsive to valsartan alone. Journal of Hypertension. 2001; 19: 2097–2104.

[3] Zhang S, Yu B, Li L, Du Z, Guan Z. Randomized, double-blinded trial evaluation of valsartan/hydrochlorothiazide combination therapy in mild to moderate essential hypertension in north-east China. The Journal of International Medical Research. 2008; 36: 630–637.

[4] Nash DT, Crikelair N, Zappe D. Achieving BP goals with valsartan and HCTZ alone and in combination: pooled analysis of two randomized, double-blind, placebo-controlled studies. Current Medical Research and Opinion. 2008; 24: 2617–2626.

[5] Duprez DA, Weintraub HS, Cushman WC, Purkayastha D, Zappe D, Samuel R, *et al*. Effect of valsartan, hydrochlorothiazide, and their combination on 24-h ambulatory blood pressure response in elderly patients with systolic hypertension: a ValVET substudy. Blood Pressure Monitoring. 2011; 16: 186–196.

[6] Allemann Y, Fraile B, Lambert M, Barbier M, Ferber P, Izzo JL, Jr. Efficacy of the combination of amlodipine and valsartan in patients with hypertension uncontrolled with previous monotherapy: the Exforge in Failure after Single Therapy (EX-FAST) study. Journal of Clinical Hypertension. 2008; 10: 185–194.

[7] Smith TR, Glazer RD, Koren MJ, Wernsing M, Zhang Y. Combination therapy with amlodipine/valsartan in essential hypertension: a 52-week, randomised, open-label, extension study. International Journal of Clinical Practice. 2010; 64: 1367–1374.

[8] Zhu D, Yang K, Sun N, Gao P, Wang R, Grosso A, *et al*. Amlodipine/valsartan 5/160 mg versus valsartan 160 mg in Chinese hypertensives. International Journal of Cardiology. 2013; 167: 2024–2030.

[9] Ruilope LM, Malacco E, Khder Y, Kandra A, Bönner G, Heintz D. Efficacy and tolerability of combination therapy with valsartan plus hydrochlorothiazide compared with amlodipine monotherapy in hypertensive patients with other cardiovascular risk factors: the VAST study. Clinical Therapeutics. 2005; 27: 578–587.

[10] Lacourcière Y, Poirier L, Hebert D, Assouline L, Stolt P, Rehel B, *et al*. Antihypertensive efficacy and tolerability of two fixed-dose combinations of valsartan and hydrochlorothiazide compared with valsartan monotherapy in patients with stage 2 or 3 systolic hypertension: an 8-week, randomized, double-blind, parallel-group trial. Clinical Therapeutics. 2005; 27: 1013–1021.

[11] Mallion JM, Carretta R, Trenkwalder P, Martinez JF, Tykarski A, Teitelbaum I, *et al*. Valsartan/hydrochlorothiazide is effective in hypertensive patients inadequately controlled by valsartan monotherapy. Blood Pressure. Supplement. 2003; 1: 36–43.

[12] Weir MR, Smith DH, Neutel JM. Valsartan alone or with a diuretic or ACE inhibitor as treatment for African American hypertensives: relation to salt intake. American Journal of Hypertension. 2001; 14: 665-671.

[13] Toyoda S, Inami S, Kato T, Tsukada K, Nakamoto A, Kikegawa Y, *et al*. Choice of Antihypertensive Combination Therapy Based on Daily Salt Intake. The American Journal of the Medical Sciences. 2015; 350: 160–166.

[14] Saito I, Saruta T, ADVANCE-Combi Study Group. Controlled release nifedipine and valsartan combination therapy in patients with essential hypertension: the adalat CR and valsartan cost-effectiveness combination (ADVANCE-combi) study. Hypertension Research. 2006; 29: 789–796.

[15] Zappe D, Papst CC, Ferber P, PROMPT Investigators. Randomized study to compare valsartan +/- HCTZ versus amlodipine +/- HCTZ strategies to maximize blood pressure control. Vascular Health and Risk Management. 2009; 5: 883–892.

[16] Segura J, Praga M, Campo C, Rodicio JL, Ruilope LM. Combination is better than monotherapy with ACE inhibitor or angiotensin receptor antagonist at recommended doses. Journal of the Renin-Angiotensin-Aldosterone System. 2003; 4: 43–47.

[17] Alici G, Aliyev F, Bellur G, Okcun B, Türkoğlu C, Karpuz H. Effect of seven different modalities of antihypertensive therapy on pulse pressure in patients with newly diagnosed stage I hypertension. Cardiovascular Therapeutics. 2009; 27: 4–9.

[18] Sison J, Assaad-Khalil SH, Najem R, Kitchlew AR, Cho B, Ueng KC, *et al*. Real-world clinical experience of amlodipine/valsartan and amlodipine/valsartan/hydrochlorothiazide in hypertension: the EXCITE study. Current Medical Research and Opinion. 2014; 30: 1937–1945.

[19] Huang J, Sun NL, Hao YM, Zhu JR, Tu Y, Curt V, *et al*. Efficacy and tolerability of a single-pill combination of amlodipine/valsartan in Asian hypertensive patients not adequately controlled with valsartan monotherapy. Clinical and Experimental Hypertension. 2011; 33: 179–186.

[20] Fujiwara T, Hoshide S, Tomitani N, Kanegae H, Kario K. Comparative effects of valsartan plus cilnidipine or hydrochlorothiazide on nocturnal home blood pressure. Journal of Clinical Hypertension. 2021; 23: 687–691.

[21] Ofili EO, Oparil S, Giles T, Pitt B, Purkayastha D, Hilkert R, *et al*. Moderate versus intensive treatment of hypertension using amlodipine/valsartan and with the addition of hydrochlorothiazide for patients uncontrolled on angiotensin receptor blocker monotherapy: results in racial/ethnic subgroups. Journal of the American Society of Hypertension. 2011; 5: 249–258.

[22] Pool JL, Glazer R, Weinberger M, Alvarado R, Huang J, Graff A. Comparison of valsartan/hydrochlorothiazide combination therapy at doses up to 320/25 mg versus monotherapy: a double-blind, placebo-controlled study followed by long-term combination therapy in hypertensive adults. Clinical Therapeutics. 2007; 29: 61–73.

[23] Giles TD, Weber MA, Basile J, Gradman AH, Bharucha DB, Chen W, *et al*. Efficacy and safety of nebivolol and valsartan as fixed-dose combination in hypertension: a randomised, multicentre study. Lancet. 2014; 383: 1889–1898.

[24] Benz JR, Black HR, Graff A, Reed A, Fitzsimmons S, Shi Y. Valsartan and hydrochlorothiazide in patients with essential hypertension. A multiple dose, double-blind, placebo controlled trial comparing combination therapy with monotherapy. Journal of Human Hypertension. 1998; 12: 861–866.

[25] Shi R, Liu K, Shi D, Liu Q, Chen X. Effects of Amlodipine and Valsartan on Blood Pressure Variability and Pulse Wave Velocity in Hypertensive Patients. The American Journal of the Medical Sciences. 2017; 353: 6–11.

[26] Farag SM, Rabea HM, Mahmoud HB. Effect of Amlodipine/Valsartan Versus Nebivolol/Valsartan Fixed Dose Combinations on Peripheral and Central Blood Pressure. High Blood Pressure & Cardiovascular Prevention. 2018; 25: 407–413.

[27] Ahn Y, Kim Y, Chang K, Kim W, Rhee MY, Cha KS, *et al*. A multicenter, randomized, and double-blind phase IV clinical trial to compare the efficacy and safety of fixed-dose combinations of amlodipine orotate/valsartan 5/160 mg versus valsartan/hydrochlorothiazide 160/12.5 mg in patients with essential hypertension uncontrolled by valsartan 160 mg monotherapy. Medicine. 2018; 97: e12329.

[28] Everett BM, Glynn RJ, Danielson E, Ridker PM, Val-MARC Investigators. Combination therapy versus monotherapy as initial treatment for stage 2 hypertension: a prespecified subgroup analysis of a community-based, randomized, open-label trial. Clinical Therapeutics. 2008; 30: 661–672.

[29] Zappe DH, Palmer BF, Calhoun DA, Purkayastha D, Samuel R, Jamerson KA. Effectiveness of initiating treatment with valsartan/hydrochlorothiazide in patients with stage-1 or stage-2 hypertension. Journal of Human Hypertension. 2010; 24: 483–491.

[30] Ofili EO, Zappe DH, Purkayastha D, Samuel R, Sowers JR. Antihypertensive and metabolic effects of Angiotensin receptor blocker/diuretic combination therapy in obese, hypertensive African American and white patients. American Journal of Therapeutics. 2013; 20: 2–12.

[31] Geiger H, Barranco E, Gorostidi M, Taylor A, Zhang X, Xiang Z, *et al*. Combination therapy with various combinations of aliskiren, valsartan, and hydrochlorothiazide in hypertensive patients not adequately responsive to hydrochlorothiazide alone. Journal of Clinical Hypertension. 2009; 11: 324–332.

[32] Flack JM, Calhoun DA, Satlin L, Barbier M, Hilkert R, Brunel P. Efficacy and safety of initial combination therapy with amlodipine/valsartan compared with amlodipine monotherapy in black patients with stage 2 hypertension: the EX-STAND study. Journal of Human Hypertension. 2009; 23: 479–489.

[33] Chrysant SG, Wombolt DG, Feliciano N. Long-term efficacy, safety, and tolerability of valsartan and hydrochlorothiazide in patients with essential hypertension. Current Therapeutic Research. 1998; 59: 762–772.

[34] Yu B. Clinical observation of valsartan combined with amlodipine in the treatment of essential hypertension. Strait Pharmaceutical Journal. 2011; 23: 190–192.
